# Barley RIC157, a potential RACB scaffold protein, is involved in susceptibility to powdery mildew

**DOI:** 10.1007/s11103-022-01329-x

**Published:** 2022-12-23

**Authors:** Stefan Engelhardt, Adriana Trutzenberg, Michaela Kopischke, Katja Probst, Christopher McCollum, Johanna Hofer, Ralph Hückelhoven

**Affiliations:** Phytopathology, TUM School of Life Sciences, Technical University of Munich, Emil- Ramann-Str.2, 85354 Freising-Weihenstephan, Germany

**Keywords:** CRIB-domain, GTPase, Susceptibility, Powdery mildew, Barley, Scaffold

## Abstract

**Key message:**

CRIB motif-containing barley RIC157 is a novel ROP scaffold protein that interacts directly with barley RACB, promotes susceptibility to fungal penetration, and colocalizes with RACB at the haustorial neck.

**Abstract:**

Successful obligate pathogens benefit from host cellular processes. For the biotrophic ascomycete fungus *Blumeria hordei* (*Bh*) it has been shown that barley RACB, a small monomeric G-protein (ROP, Rho of plants), is required for full susceptibility to fungal penetration. The susceptibility function of RACB probably lies in its role in cell polarity, which may be co-opted by the pathogen for invasive ingrowth of its haustorium. However, how RACB supports fungal penetration success and which other host proteins coordinate this process is incompletely understood. RIC (ROP-Interactive and CRIB-(Cdc42/Rac Interactive Binding) motif-containing) proteins are considered scaffold proteins which can interact directly with ROPs via a conserved CRIB motif. Here we describe a previously uncharacterized barley RIC protein, RIC157, which can interact directly with RACB *in planta*. We show that, in the presence of constitutively activated RACB, RIC157 shows a localization at the cell periphery/plasma membrane, whereas it otherwise localizes to the cytoplasm. RIC157 appears to mutually stabilize the plasma membrane localization of the activated ROP. During fungal infection, RIC157 and RACB colocalize at the penetration site, particularly at the haustorial neck. Additionally, transiently overexpressed RIC157 renders barley epidermal cells more susceptible to fungal penetration. We discuss that RIC157 may promote fungal penetration into barley epidermal cells by operating probably downstream of activated RACB.

**Supplementary Information:**

The online version contains supplementary material available at 10.1007/s11103-022-01329-x.

## Introduction

Plants have developed a multilayered immunity to defend microbial invasion. This consists of pre-formed barriers and induced defenses that are based on the receptor-mediated recognition of microbe-derived and endogenous elicitors (Ngou et al. 2022; Rzemieniewski and Stegmann 2022). Except for necrotrophs, invading microbes rely to different extents on a living host to establish an infection. With the help of secreted effectors, pathogens undermine plant immune reactions and influence the host metabolism to render the micro-environment more favourable for their own proliferation (Białas et al. 2018; Han and Kahmann 2019). The co-evolution between microbial effectors and their specific host target molecules in a symbiotic relationship, be it mutualistic, commensalistic or parasitic, often leads to an increase in microbial specialization on a certain plant host. In genomes of obligate biotrophic pathogens, in particular cereal powdery mildew fungi, the amount of genes encoding for metabolic enzymes is massively reduced. This reduction coincided with the proliferation of putative effector genes and transposable elements (Spanu et al. 2010; Wicker et al. 2013; Frantzeskakis et al. 2018). Plant targets of these effectors are not necessarily involved in resistance mechanisms, but also in cellular processes that can support the susceptibility towards the invading pathogen. With the current possibilities to utilize a plethora of different breeding technologies, durable crop resistance based on the control of these susceptibility gene functions is within reach (Dangl et al. 2013; Engelhardt et al. 2018).

Powdery mildew fungi infect a huge variety of monocot and dicot plants causing massive yield losses in crops. The ascomycete fungus *Blumeria hordei* (*Bh*) is the specific causal agent of the agronomically important powdery mildew disease on barley (*Hordeum vulgare*) (Jørgensen and Wolfe 1994). As an obligate biotrophic parasite, *Bh* requires living epidermal cells to complete its life cycle. Airborne conidia germinate on the leaf surface and form appressoria to penetrate the cuticle and the cell wall with the help of turgor pressure and the release of cell wall-degrading enzymes (McKeen and Rimmer 1973; Schulze-Lefert and Vogel 2000; Hückelhoven and Panstruga 2011). A successful fungal infection is characterised by the formation of a haustorium inside the host cell, which is essential for nutrient uptake and effector proteins delivery (Hahn and Mendgen 2001; Voegele et al. 2001; Panstruga and Dodds 2009). The haustorium is separated from the host cytosol by the extrahaustorial matrix and surrounded by the extrahaustorial membrane (EHM). The EHM is continuous with the plant plasma membrane, but differs functionally and biochemically from it (Koh et al. 2005; Inada and Ueda 2014; Kwaaitaal et al. 2017). It is feasible to imagine pathogen-triggered active contributions of the plant to accommodate the fungal haustorium.

ROPs (RHO (RAS homologue) of plants, or RACs, for rat sarcoma (RAS)-related C3 botulinum toxin substrate) form a unique subfamily of small monomeric RHO GTPases in plants (Brembu et al. 2006; Engelhardt et al. 2020). G-proteins are paradigms of molecular switches due to their ability to bind and hydrolyze GTP, accompanied by conformational changes allowing to interact directly with specific proteins. The GTP-bound form represents the activated state, and a plasma membrane association of ROP-GTP via posttranslational lipid modifications is required for downstream signaling (Yalovsky 2015). Upon GTP hydrolysis, GDP-bound or nucleotide-free ROPs are inactive regarding downstream signaling. The cycling between activated and inactive states needs to be spatiotemporally controlled by regulatory partners. Guanine nucleotide exchange factors (GEFs) positively regulate ROP activity by facilitating the GDP/GTP exchange. In plants, three different sorts of ROP GEFs can be distinguished based on their particular GEF domain: PRONE (plant-specific Rop nucleotide exchanger), DHR2 (DOCK homology region 2, found in SPIKE1) and a less well characterized DH-PH domain (B-cell lymphoma homology-pleckstrin homology) described in a plant homolog of human SWAP70 (Berken et al. 2005; Meller et al. 2005; Gu et al. 2006; Basu et al. 2008; Yamaguchi and Kawasaki 2012; Yamaguchi et al. 2012; He et al. 2018). The interaction of ROPs with a GTPase activating protein (GAP) enhances the intrinsic GTP hydrolysis activity, followed by ROP inactivation (Berken and Wittinghofer 2008). Besides their putative involvement in ROP recycling, guanine nucleotide dissociation inhibitors (GDIs) bind and sequester inactive ROPs in the cytoplasm and are therefore considered negative regulators of ROP activity (Klahre et al. 2006; Boulter and Garcia-Mata 2010). ROPs are involved in the regulation of a multitude of cellular processes. For instance, the cytoskeleton organisation and, consequentially, cell shape and function is subject to ROP GTPase control (Chen and Friml 2014). In *Arabidopsis thaliana* xylem vessels, *At*ROP11 signaling promotes cell wall apposition and shapes cell wall pit boundaries (Sugiyama et al. 2019). Different ROPs are involved in polar cell growth and even function antagonistically during the generation of *Arabidopsis thaliana* pavement cells (Craddock et al. 2012). Besides cell polarisation and cytoskeleton organisation, ROPs have been also implicated in membrane trafficking and auxin signaling (Yalovsky et al. 2008; Wu et al. 2011). *Os*Rac1 from rice (*Oryza sativa*) enhances cell division by regulating *Os*MAPK6, thereby promoting rice grain yield (Zhang et al. 2019). It has also been demonstrated that *Os*RAC1 regulates immune-related processes like ROS production, defense gene expression and cell death. To achieve this, *Os*Rac1 forms specific immune receptor complexes containing either *Os*CERK1, a chitin pattern recognition coreceptor, or plasma membrane-localized Pit, a nucleotide binding-leucine rich repeat resistance (NLR) protein against the rice blast fungus *Magnaporthe oryzae* (Akamatsu et al. 2021). *Os*Rac1 is activated either via *Os*RacGEF1 upon perception of fungal-derived chitin by *Os*CEBiP and *Os*CERK1 (Akamatsu et al. 2013), or via DOCK family GEF *Os*SPK1 that associates with activated Pit (Kawano et al. 2010; Kawano et al. 2014; Wang et al. 2018). Recently, the NLR protein PID3 (Zhou et al. 2019) was shown to activate *Os*Rac1 also via *Os*SPK1 (Yu et al. 2021), which opens up the possibility of *Os*Rac1 being a downstream hub of other rice NLR proteins.

In the barley-powdery mildew interaction, several barley proteins involved in ROP signaling or ROP activity regulation have been shown to influence fungal penetration success. In this context, the barley ROP protein RACB has been shown to be required for full susceptibility towards fungal infection (Schultheiss et al. 2002; Schultheiss et al. 2003; Schnepf et al. 2018). The expression of a constitutively activated GTP-bound RACB promoted fungal penetration success into barley epidermal cells, whereas RACB silencing by RNA interference (RNAi) renders epidermal cells less susceptible. In absence of the pathogen, RACB’s elemental function appears to be the regulation of cell polarization processes, because stable RACB silencing affects stomatal subsidiary cells and root hair development (Scheler et al. 2016). Recently, barley PRONE-GEF14 was demonstrated to be transcriptionally regulated during fungal infection and, by directly interacting, likely involved in RACB activation (Trutzenberg et al. 2022). Two interactors of activated RACB have been described as negative regulators of RACB function in susceptibility. First, the Microtubule-Associated ROP-GAP1 (MAGAP1) is recruited to the cell periphery by activated RACB and limits susceptibility to powdery mildew likely by enhancing RACB’s GTP-hydrolizing activity (Hoefle et al. 2011). Activated RACB also interacts with the cytoplasmic ROP binding kinase1 (RBK1) in vivo and enhances its kinase activity in vitro (Huesmann et al. 2012). Transient silencing of RBK1 or RBK1-interacting protein SKP1 (type II S-phase kinase1-associated protein) suggested that RBK1 negatively regulates RACB protein stability and hence rather acts in disease resistance (Reiner et al. 2016). A highly conserved lysine residue that is targeted for ubiquitination further supports the idea of proteasomal regulation of RACB (Weiß et al. 2022).

ROPs need to activate or deactivate downstream executors (also called ROP effectors), to regulate cellular processes. The interaction to some of these executors is often indirect and achieved via scaffold proteins bridging the activated ROPs to their signal destination targets. Some ROP scaffold proteins like RACK1 (Receptor for Activated C-Kinase 1), ICR/RIPs (Interactor of Constitutive Active ROP/ROP Interactive Partners) and RICs (ROP-Interactive and CRIB-(Cdc42/Rac Interactive Binding) motif-containing) have been described so far in detail. Rice RACK1 interacts with several proteins in the *Os*Rac1 immune complex supporting a role in rice innate immunity (Nakashima et al. 2008). ICR/RIPs are required for cell polarity, vesicle trafficking, and polar auxin transport (Lavy et al. 2007; Hazak et al. 2010). In barley, RIPa interacts with the ROP GTPase RAC1 and organizes microtubule arrays in concert with MAGAP1 (Hoefle et al. 2020). Barley RIPb interacts directly with RACB and enhances disease susceptibility towards powdery mildew (McCollum et al. 2020). RIC proteins, another class of scaffold proteins in ROP signaling, share a highly conserved CRIB motif (Cdc42/Rac Interactive Binding motif, Burbelo et al. 1995), which is essential for the direct interaction with ROPs (Wu et al. 2001). The CRIB domain is also present in a subset of ROP GAPs such as barley MAGAP1 (Schaefer et al. 2011; Hoefle et al. 2011). In barley, the knowledge about RIC protein functions is quite limited. Barley RIC nomenclature follows the number of amino acids in the predicted open reading frames. RIC171 has been shown to interact directly with RACB and to increase fungal penetration efficiency in barley epidermal cells upon overexpression. Activated RACB recruits RIC171 to the cell periphery and, in the presence of *Bh*, RIC171 accumulated at the haustorial neck close to the penetration site (Schultheiss et al. 2008, Hückelhoven and Panstruga 2011). In *Arabidopsis thaliana* (*At*), 11 different RIC proteins have been identified, that do not share common sequence homology outside their CRIB domain (Wu et al. 2001). By directly interacting with *At*ROPs, *At*RIC proteins are involved in numerous cellular processes. During salt stress, *At*ROP2 regulates microtubule organisation in an *At*RIC1-dependent manner (Li et al. 2017). *At*RIC1 also interacts with *At*ROP6 in pavement cells to enhance the ordering of cortical microtubules upon hormonal signals (Fu et al. 2009) and is involved in cell elongation during pavement cell morphogenesis (Higaki et al. 2017). *At*RICs counteract each other to a certain extent as well, as seen with *At*ROP1-interacting *At*RIC3 and *At*RIC4 during pollen tube growth. *At*RIC3 regulates calcium influx and triggers actin depolymerisation, whereas *At*RIC4 enhances actin polymerisation (Gu et al. 2005). Light-induced stomatal opening is regulated via the *At*ROP2-*At*RIC7 pathway. *At*ROP2 and *At*RIC7 are likely to impinge on vesicular trafficking by inhibiting the exocyst subunit *At*Exo70B1, which results in a diminished stomatal opening (Hong et al. 2016). These examples emphasize the importance of ROP proteins as signaling hubs for developmental processes as well as the role of RIC proteins in finetuning specific cellular responses.

Here we show results on barley RIC157, a CRIB domain-containing protein that interacts directly with RACB in yeast and *in planta*. Overexpression of RIC157 increases the powdery mildew penetration efficiency in barley leaf epidermal cells. Cytosolic RIC157 is recruited to the cell periphery specifically by activated RACB, where it appears to stabilize the plasma membrane association of activated RACB. Both proteins co-localize at the haustorial neck during the compatible interaction with *Bh* suggesting a possible role of a RACB-RIC157 signaling module in promoting susceptibility to fungal penetration.

## Materials and methods

### Plant and fungal growth conditions

Wildtype barley (*Hordeum vulgare*, cultivar Golden Promise) was cultivated in long day conditions (16 h day light, 8 h darkness) at a temperature of 18 °C with a relative humidity of 65% and a light intensity of 150 µmol s^− 1^ m^− 2^.

The biotrophic powdery mildew fungus *Blumeria graminis* f.sp. *hordei* A6 was used in all experiments. It was cultivated and propagated on barley Golden Promise under the same conditions described above.

### Cloning of constructs

Via a Two-Step Gateway cloning approach, in a first PCR *RIC157* (HORVU.MOREX.r3.6HG0565950) was amplified from a barley (cultivar Golden Promise) cDNA pool prepared from leaves and epidermal peels using gene-specific primers RIC157_GW_for and RIC157_GW_rev + STOP (Suppl. Table 1) creating an incomplete Gateway attachment site overhang. A second PCR using primers attB1 and attB2 completed the attachment sites. To create a Gateway entry clone of *RIC157*, the amplified product was recombined into pDONR223 (Invitrogen) via BP-reaction using Gateway BP Clonase™ II according to manufacturer’s instruction (Thermo Fisher Scientific). To clone *RIC157ΔCRIB*, both fragments upstream and downstream of CRIB motif were amplified seperately using primers RIC157_GW_for and RIC157delCRIB_rev for PCR1, RIC157delCRIB_for and RIC157_GW_rev + STOP for PCR2, creating overlapping overhangs. In PCR3 both fragments together with primers RIC157_GW_for and RIC157_GW_rev + STOP completed *RIC157ΔCRIB* with incomplete Gateway attachment sites overhangs. Completetion of attachment sites and creating an entry clone in pDONR223 was done as described above. For cloning entry constructs of *RACB* variants, primers RACB_GW_for and RACB_GW_rev were used to amplify *RACB* from previously described constructs (Schultheiss et al. 2003) and cloned into pDONR223 via BP as described abobe. To clone *RACB(D121N)*, a site-directed mutagenesis using primers RACB_D121N_fw and RACB_D121N_rv was performed according to QuikChange® Site-Directed Mutaganesis Protocol (Stratagene).

For RNA interference (RNAi) silencing of *RIC157* in barley, we PCR-amplified two RIC157 fragments, a 97 bp fragment with primers RIC157_RNAi_NotI_for and RIC157_RNAi_EcoRI_rev containing *Not*I and *Eco*RI restriction sites, and a 324 bp fragment with primers RIC157_RNAi_EcoRI_for and RIC157_RNAi_XbaI_rev containing *Eco*RI and *Xba*I restriction sites. After restriction digest of all sites, ligating into pIPKTA38 via *Not*I and *Xba*I sites we created a *RIC157* RNAi entry construct lacking the CRIB motif nucleotide sequence to prevent off-target silencing of other CRIB-domain containing RNAs. The *RIC157* RNAi sequence was then cloned via LR reaction using Gateway LR Clonase™ II according to manufacturer’s instruction (Thermo Fisher Scientific) into RNAi expression plasmid pIPKTA30N to create a double-strand RNAi expression construct (Douchkov et al. 2005).

For Yeast-2-Hybrid expression clones, entry clones of *RIC157* and *RACB* variants were introduced into prey plasmid pGADT7-GW and pGBKT7-GW via LR reaction using Gateway LR Clonase™ II according to manufacturer’s instruction (Thermo Fisher Scientific). pGADT7-GW and pGBKT7-GW have been modified from pGADT7 and pGBKT7 (Clontech) into a Gateway-compatible form using Gateway™ Vector Conversion System (Thermo Fisher Scientific). To create *RACB* variants lacking C-terminal prenylation sequence, a premature STOP-Codon was introduced by site-directed mutagenesis as described above using primers delCSIL_for and delCSIL_rev.

For localization and overexpression studies in barley, *RIC157* and *RACB* variants in pDONR223 were used as entry constructs to clone them into various pGY1-based CaMV35S promoter-driven expression vectors (Schweizer et al. 1999) via LR reaction as described above. Empty pGY1 (encoding for no tag) was rendered Gateway-compatible via Gateway™ Vector Conversion System (Thermo Fisher Scientific). To create expression vectors for proteins C- or N-terminally tagged by GFP or mCherry, Gateway Reading Frame Cassettes for C- and N-terminal fusions, respectively, were integrated into a pGY1-plasmid backbone upon XbaI digestion and combined at 5′ or 3′ with sequences of monomeric GFP or mCherry. Cloning procedure was performed using In-Fusion HD cloning kit (Takara Bio USA). Constructs for GFP and mCherry upstream or downstream of the Gateway cassette were amplified using primers GW_RfA_mCherry-F, GW_RfA_meGFP-F, GW_RfA_Xba-R, GW_Xba_RfB-F, GW_RfB-R, meGFP-STP-F, mCherry-STP-F, XFP-noSTP_Xba-F, XFP-noSTP-R, meGFP-noSTP-R, mCherry-STP_Xba-R and meGFP-STP_Xba-R.

### Barley epidermal cell transformation and penetration efficiency assessment

For transient overexpression in barley, primary leaf epidermal cells of 7 days-old plants were transformed using biolistic bombardment with 1 μm gold particles that were coated with 2 µg of each test plasmid and additionally with 1 µg of a cytosolic transformation marker. After mixing the gold particles with plasmid combinations, CaCl_2_ (0.5 M final concentration) and 3.5 µl of 2 mg/ml Protamine (Sigma) were added to each sample. The gold particle solution was incubated at room temperature for 30 min, washed twice with 500 µl Ethanol (first 70%, then 100%) and eventually dissolved in 6 µl 100% ethanol per biolistic transformation. After shooting, leaves were incubated at 18 °C. For localization experiments, leaves were analysed 2 days after transformation. For FRET-FLIM analysis of RACB-RIC157 interaction, barley primary leaves of 7 days-old plants were transiently transformed. Therefore, 2 µg of mCherry-RACB and 1 µg meGFP-RIC157 containing plasmids were coated on gold particles for biolistic transformation of single barley epidermal cells.

For inoculation with *Bh*, fungal spores were manually blown in a closed infection device over transformed leaves either 6 h after transformation (for microscopic analyses 16 h after inoculation) or 1 day after transformation (to check penetration efficiency 48 h after inoculation).

To analyse penetration efficiency, a transient assay system based on a cytosolic GUS marker was used as decribed previously (Schweizer et al. 1999). The reporter gene construct pUbiGUSPlus was a gift from Claudia Vickers (Addgene plasmid # 64,402; http://n2t.net/addgene:64402; RRID: Addgene_64402, Vickers et al. 2003). Additionally, to overexpression or RNAi silencing constructs, each barley leaf was co-transformed with pUbiGUSPlus. 48 h after *Bh* inoculation, leaves were submerged in GUS staining solution (0.1 M Na_2_HPO_4_/NaH_2_PO_4_ pH 7.0, 0.01 M EDTA, 0.005 M Potassium hexacyanoferrat (II), 0.005 M Potassium hexacyanoferrat (III), 0.1% (v/v) Triton X-100, 20% (v/v) Methanol, 0.5 mg/mL 1.5-bromo-4-chloro-3-indoxyl-β-D-glucuronic acid). For the solution to enter the leaf interior, a vacuum was applied. The leaves were incubated at 37 °C over night in GUS staining solution and subsequently for at least 24 h in 70% Ethanol. Fungal structures were stained with ink-acetate solution (10% ink, 25% acetic acid). Transformed cells were identified after GUS staining with light microscopy. An established haustorium was considered a successful penetration and for each sample at least 50 interactions were analysed. Barley epidermal cells transformed with the empty expression plasmid were used as negative control.

### Yeast-2-hybrid

Yeast strain AH109 was transformed with bait (pGBKT7) and prey (pGADT7) constructs by following the small scale yeast transformation protocol from Yeastmaker™ Yeast Transformation System 2 (Clontech). Upon transformation, yeast cells were plated on complete supplement medium (CSM) plates lacking leucine and tryptophan (LW) and incubated for 3 days at 30 °C. A single colony was taken to inoculate 5 mL of LW-dropout liquid medium that was incubated with shaking over night at 30 °C. The next day, 2 mL of culture was pelleted for immunoblot analyses. 7.5 µL of undiluted overnight culture (and additionally a 1:10, 1:100 and 1:1000 for control purposes) were dropped on CSM plates lacking leucine and tryptophan, and also on CSM plates lacking leucine, tryptophan and adenine. Plates were incubated for at least 3 days at 30 °C. Growth on CSM-LW plates confirmed the successful transformation of both bait and prey plasmids, while growth on CSM-LWAde plates indicated activation of reporter genes. As control for a positive and direct protein-protein interaction we routinely used murine p53 and the SV40 large T-antigen (Li and Fields 1993).

### Microscopy

Localzsation experiments were analysed on a Leica TCS SP5 confocal laser scanning microscope. The excitation laser wavelengths were 488 nm for GFP and 561 nm for RFP and mCherry, respectively. The fluorescence emission was collected from 500 to 550 nm for GFP and from 569 to 610 nm for RFP and mCherry. Barley epidermal cells were imaged via sequential scanning as z-stacks in 2 μm increments. Maximum projections of each z-stack were exported as Tiff files from the Leica LAS AF software (version 3.3.0).

Localization experiments of fluorescent protein fusions in barley epidermal cells were conducted from 24 h to 48 h after biolistic transformation.

For FRET-FLIM analysis of RACB-RIC157 interaction, the expression of the fluorophore-fusion proteins was analysed 1 day after transformation using an Olympus FluoView™3000 inverse laser scanning confocal microscope with an UPLSAPO 60XW 60x/NA 1.2/WD 0.28 water immersion objective (Olympus, Hamburg, Germany). Fluorescence of GFP was collected between 500 and 540 nm and mCherry emission was imaged between 580 and 620 nm upon excitation with 488 and 561 nm argon laser lines, respectively. For FRET-FLIM measurements the PicoQuant advanced FCS/FLIM-FRET/rapidFLIM upgrade kit (PicoQuant, Berlin, Germany) was used, comprising a 485 nm pulsed laser line for GFP excitation (pulse rate 40 mHz, laser driver: PDL 828 SEPIA II, laser: LDH-D-C-485), a Hybrid Photomultiplier Detector Assembly 40 to detect GFP fluorescence and a TimeHarp 260 PICO Time-Correlated Single Photon Counting module (resolution 25 ps) to measure photon life times. GFP fluorescence was imaged at the equatorial plane of epidermis cells to capture GFP fluorescence at the cell periphery and possibly plasma membrane. For each interaction at least 10 cells were analysed in two replicates and a minimum of 1000 photons of the brightest pixel were recorded. Decay data within a region of interest were fitted using an n-exponential reconvolution fit with model parameters n = 3 and measured instrument response function.

## Results

### Identification of RIC proteins in barley

**Fig. 1:**
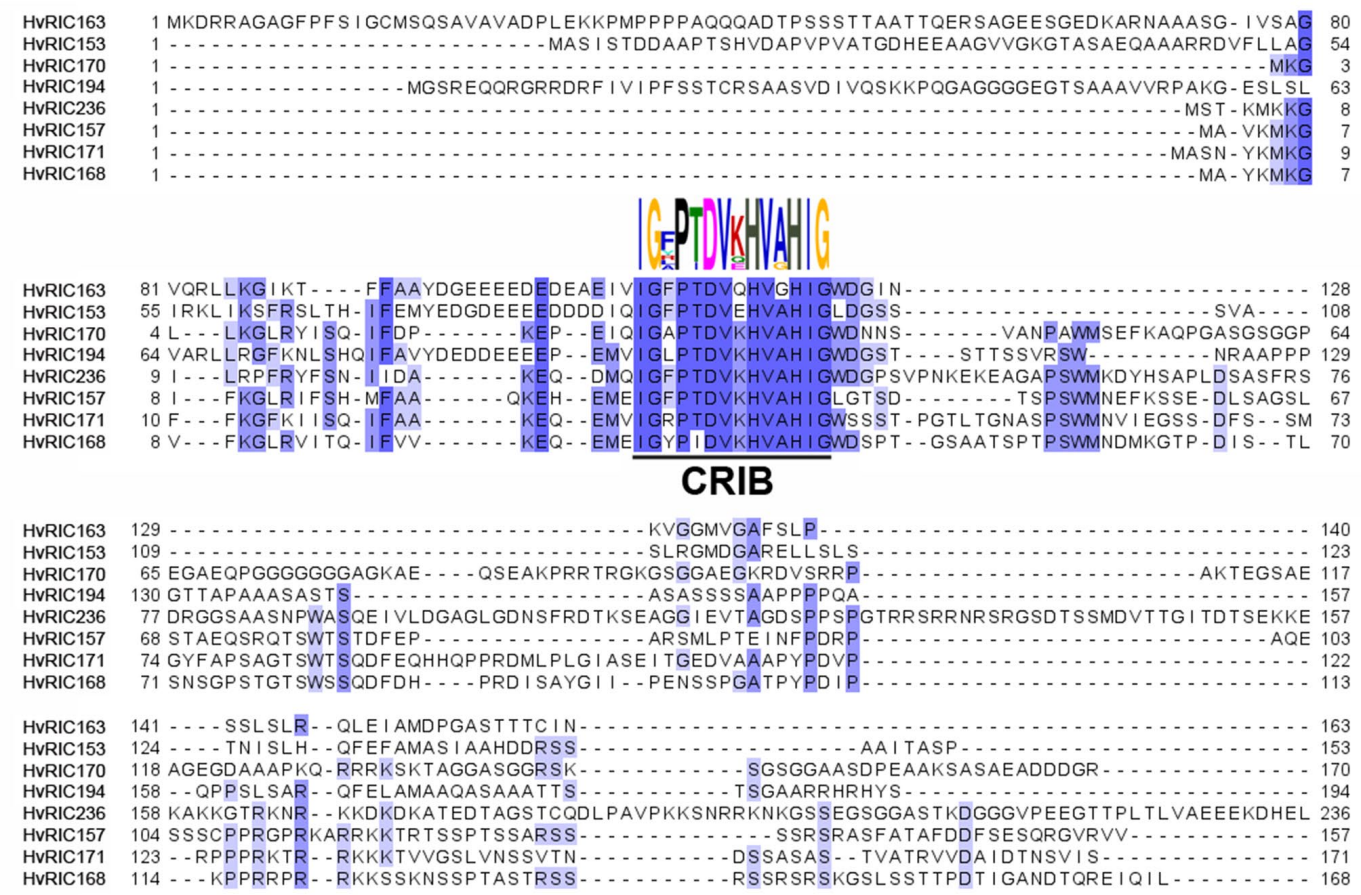
Barley RIC proteins alignment. Multiple alignment (https://www.ebi.ac.uk/Tools/msa/muscle, Edgar 2004; illustrated using Jalview 2.11.0 software) of predicted barley (*Hordeum vulgare*, *Hv*) proteins harboring CRIB domain (underlined). Intensity of blue coloured amino acids represents level of conservation (higher intensity = more conserved). CRIB domain consensus sequence of barley RIC proteins is indicated by coloured amino acid sequence above the alignment (http://meme-suite.org/tools/meme)

Individual RIC proteins typically show a lack of primary sequence homology to other proteins in the database outside their CRIB domain (Wu et al. 2001). This highly conserved motif has been shown to interact directly with activated small RHO GTPases (Burbelo et al. 1995; Aspenström 1999). In order to identify additional RIC proteins in barley, we performed a BLAST search using the nucleotide sequence of the CRIB motif of previously described RACB interacting protein RIC171 (Schultheiss et al. 2008) against the 2020 annotation of all barley coding sequences (Barley all CDS Morex v3.0 2020, https://galaxy-web.ipk-gatersleben.de/, Cock et al. 2015). Besides RIC171 (HORVU.MOREX.r3.2HG0198220), we identified another seven proteins sharing the properties of RIC proteins, and sticked initially to the concept first used in Schultheiss et al. (2008) to name them according to their predicted amino acid sequence length: RIC153 (HORVU.MOREX.r3.3HG0309900), RIC157 (HORVU.MOREX.r3.6HG0565950), RIC163 (HORVU.MOREX.r3.5HG0533430), RIC168 (HORVU.MOREX.r3.2HG0205330), RIC170 (HORVU.MOREX.r3.6HG0627790), RIC194 (HORVU.MOREX.r3.3HG0309760), RIC236 (HORVU.MOREX.r3.2HG0148570). A primary sequence alignment of all eight barley RIC proteins illustrated no general domain homologies outside the highly conserved CRIB motif (Fig. 1). Interestingly, the CRIB motif was more C-terminally located in RICs 153, 163 and 194, similar to RIC2 and RIC4 of *Arabidopsis thaliana* (Wu et al. 2001), while the other RICs (157, 168, 170, 171 and 236) contained the CRIB motif closer to their N-terminal end. However, additional highly conserved domains shared by all members of the barley RIC protein family could not be identified, although we noted an area rich in positively charged amino acids close to the C-terminal end in RIC proteins 157, 168, 170, 171 and 236.

RACB-mediated susceptibility towards powdery mildew is determined in barley leaf epidermal cells. Hence, in order to unravel downstream signaling components of RACB, we focused on barley leaf-expressed RIC proteins. Using online available gene expression databases based on RNAseq analysis (https://ics.hutton.ac.uk/barleyrtd/blast_page.html, Mascher et al. 2021), we identified five leaf-expressed *RIC* genes *RIC153*, *RIC157*, *RIC163*, *RIC194* and previously published *RIC171* (Schultheiss et al. 2008) of which *RIC157*, *RIC163*, *RIC171* and *RIC194* were expressed in epidermal cells. Despite little sequence conservation between RIC proteins outside their CRIB domain, we compared the amino acid sequences of barley (*Hordeum vulgare*), *Arabidopsis thaliana*, and rice (*Oryza sativa*) RIC proteins phylogenetically (Suppl. Figure 1) using an available online software (http://doua.prabi.fr/software/seaview) to perform a MUSCLE alignment followed by calculating a maximum likelihood tree via PhyML. We identified two distinct clades each with two leaf epidermis-expressed RIC proteins, demonstrating structural and likely functional diversities among leaf epidermis-expressed RIC proteins. In clade II, RIC157 clustered together with RIC171 indicating a close resemblance which prompted us to concentrate our efforts on the functional characterization of this particular barley RIC protein.

### RIC157 increases susceptibility of barley towards ***Bh***

**Fig. 2:**
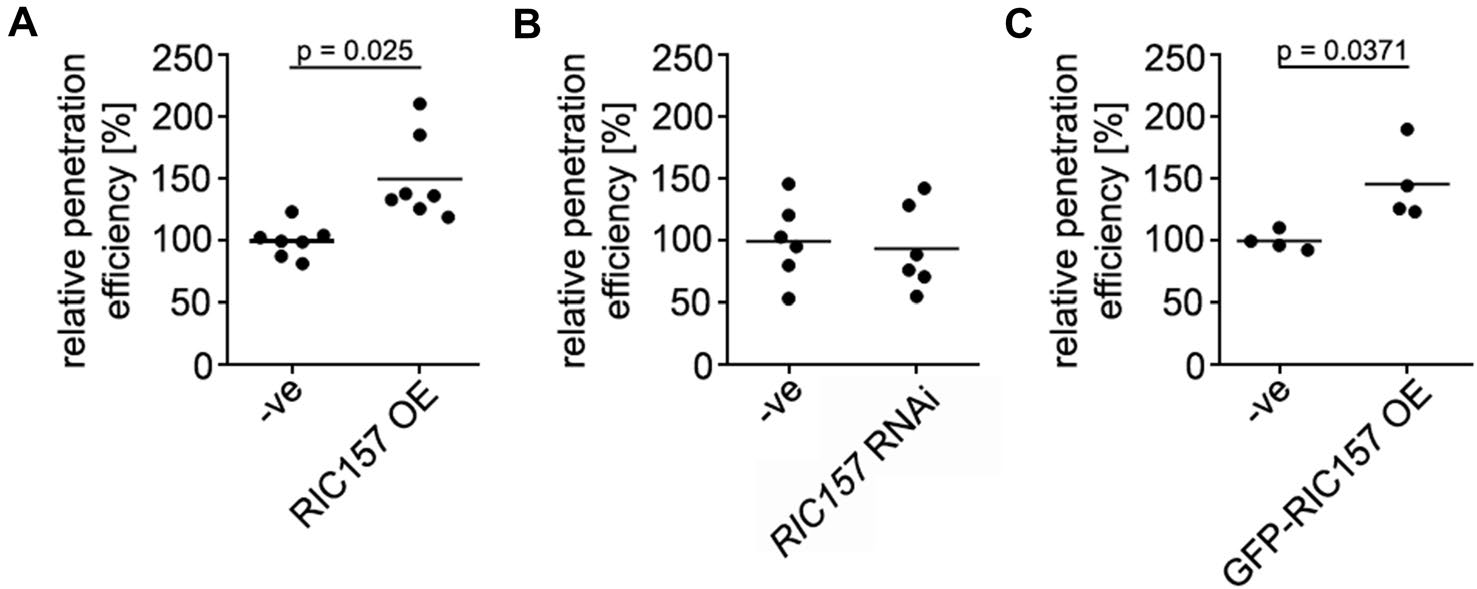
RIC157 increases susceptibility. Epidermal cells of 7-days-old primary barley leaves were transiently transformed by particle bombardment with either **A** an overexpression construct of RIC157, **B** an RNA interference construct of *RIC157*, or **C** an overexpression construct of RIC157 N-terminally tagged with GFP. Empty overexpression or RNA interference plasmids were used as respective controls (− ve). For each experimental setup a GUS construct was co-bombarded and used as transformation marker. The penetration efficiency of *Bh* into transformed barley epidermal cells was analysed 48 h after inoculation with fungal spores. Values are shown as mean of at least four independent biological replicates, relative to the mean of the control set as 100%. Statistical significance was calculated with t-test

To investigate a potential biological function of RIC157 during the barley-powdery mildew interaction, we analysed the penetration success of *Bh* on barley epidermal cells during various conditions in a series of independent experiments (Fig. 2). Single cell transient overexpression of RIC157 had a significant effect on the penetration success of *Bh* into barley epidermal cells (Fig. 2A). The susceptibility increased by about 50% compared to control treatments. We, however, did not observe a decreased fungal penetration indicative of an elevated defense upon RNA interference (RNAi)-mediated silencing of RIC157 compared to control levels (Fig. 2B). To rule out a dysfunctional RNAi construct, we confirmed the efficiency of the silencing via co-expression of a fluorescence tag-labelled RIC157 and ratiometric fluorescence measurements (Suppl. Fig. S2).

### RIC157 interacts directly with RACB in yeast and ***in planta***

**Fig. 3:**
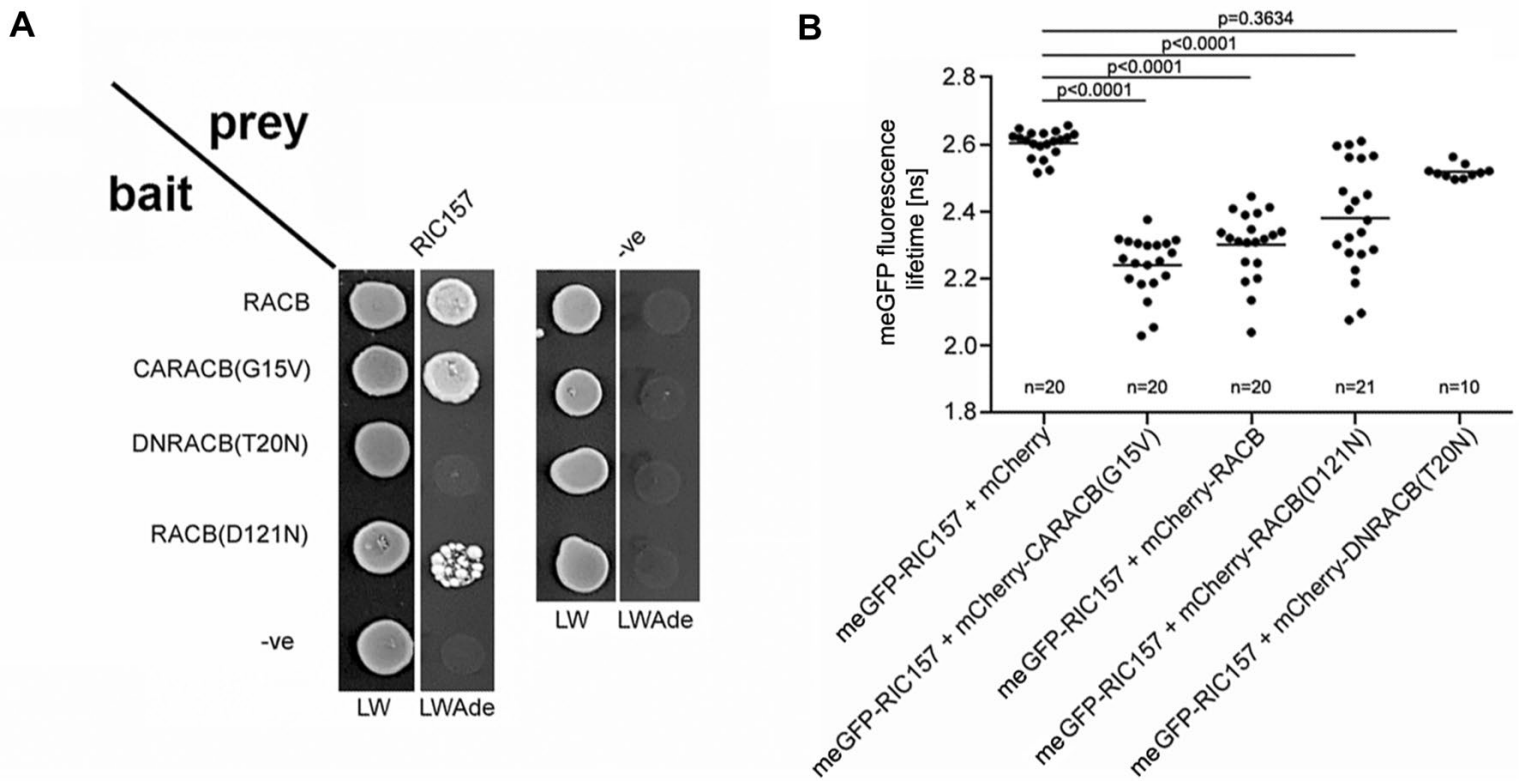
RIC157 interacts directly with RACB in yeast and *in planta*. **A** Yeast-2-Hybrid assay demonstrates direct interaction between RIC157 and RACB (lacking C-terminal CSIL domain) in yeast. Growth on LWAde medium indicates interaction between bait and prey fusion proteins. – ve denotes empty prey and bait plasmid. Photos were taken 2 days (LW) and 7 days (LWAde), respectively, after dropping. **B** Quantification of FLIM analysis confirms direct protein-protein interaction between RIC157 and RACB *in planta*. meGFP fluorescence lifetime in barley epidermal cells transiently co-expressing indicated constructs was investigated at the aequatorial plane 2d after transformation via particle bombardment. n number of cells observed. Statistical analysis was performed with one-way-Anova including Bonferroni’s multiple comparisons test (Graph Pad Prism, version 8.0.1.)

RACB can directly interact with CRIB motif-containing proteins RIC171 and MAGAP1 (Schultheiss et al. 2008; Hoefle et al. 2011), suggesting that RIC157 might directly interact with RACB in a similar fashion. We therefore conducted different approaches to assess direct protein-protein interaction between RIC157 and RACB. For these experimental approaches in yeast and *in planta* we deployed, besides the wild type, a set of specific RACB mutant forms: RACB-G15V which represents a constitutively activated (CA) GTP-bound RACB mutant; RACB-T20N that creates a dominant negative (DN) GDP-bound RACB form; RACB-D121N that is considered to possess low affinity to both GDP and GTP (Cool et al. 1999). In a targeted yeast-2-hybrid experiment in which we used truncated RACB forms lacking the C-terminal CSIL motif to prevent lipidation and plasma membrane association, we found that RIC157 directly interacts with RACB and with the constitutively activated CARACB(G15V) mutant. We also observed a certain level of interaction between RIC157 and RACB(D121N), but there was no growth of yeast colonies expressing DNRACB(T20N) (Fig. 3A).

To corroborate these initial direct interaction results *in planta*, we analysed the interaction between RACB and RIC157 via  fluorescence lifetime imaging of förster resonance energy transfer (FLIM-FRET), using in particular the monomeric enhanced green fluorescent protein (meGFP) as FRET donor and mCherry as FRET acceptor (Fig. 3B). We fused meGFP N-terminally to RIC157 and mCherry N-terminally to the different RACB forms and transiently co-expressed respective combinations in barley epidermal cells via biolistic transformation. Co-expression of meGFP-RIC157 with free mCherry resulted in an average meGFP lifetime of about 2.6 ns and is representative of a non-FRET donor-only setup. meGFP fluorescence lifetime was not significantly lower compared to the donor-only control samples in cells co-expressing meGFP-RIC157 and mCherry-DNRACB(T20N). In contrast to that, the co-expression of meGFP-RIC157 with mCherry-CARACB(G15V) led to a strong and highly significant reduction in meGFP fluorescence lifetime to approximately 2.2 ns. This meGFP fluorescence lifetime reduction demonstrates a direct protein-protein interaction between RIC157 and activated RACB *in planta*. We observed a very similar reduction in meGFP fluorescence lifetime when meGFP-RIC157 was co-expressed with mCherry-RACB suggesting an interaction of RIC157 with the wildtype form of RACB. The FLIM approach also indicated an interaction of RIC157 with the lower nucleotide affinity RACB(D121N) mutant. However, the data points ranged from no meGFP fluorescence lifetime reduction down to lifetime reductions reminiscent of mCherry-CARACB(G15V)-expressing cells. Since the D121N mutation leads to a lower nucleotide affinity, this particular result might have been provoked by different physiological cell conditions which in some cells stabilize the GDP-bound, signaling-inactive conformation and in others the GTP-bound, activated conformation of the RACB(D121N). In total, these different experimental approaches strongly support a direct protein-protein interaction between RIC157 and the activated form of RACB.

### Recruitment of RIC157 to the cell periphery by RACB

**Fig. 4:**
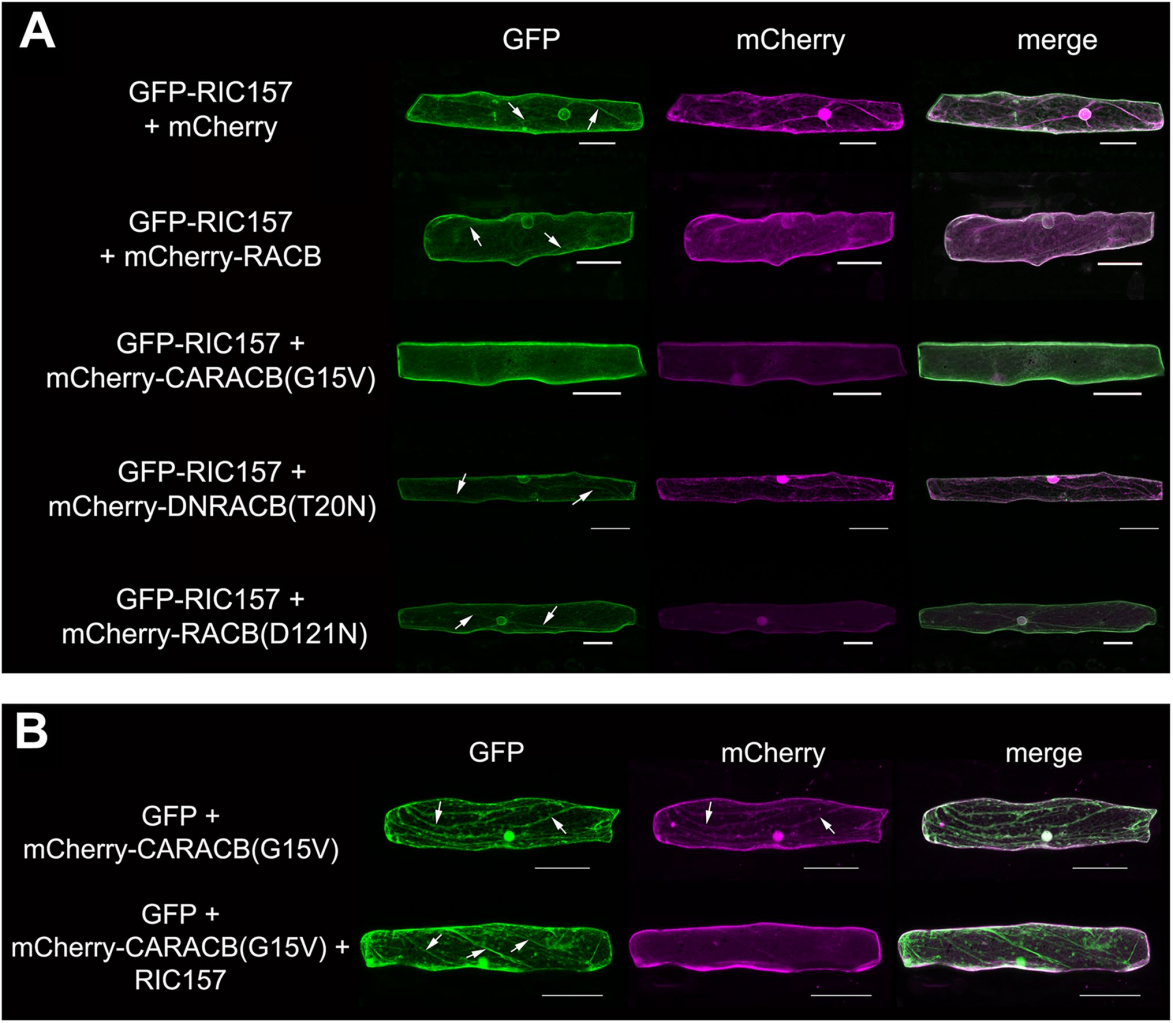
RIC157 and RACB affect each other’s localization. Confocal laser scanning microscopy of barley epidermal cells 1d after transformation via particle bombardment. **A** GFP-RIC157 localizes to the cytoplasm, but not the nucleus (upper row) and is recruited to the cell periphery exclusively by co-overexpressed mCherry-CARACB(G15V), but not by mCherry-RACB, mCherry-DNRACB(T20N) or mCherry-RACB(D121N). **B** The plasma membrane-associated localization of activated RACB (mCherry-CARACB(G15V)) becomes more exclusive in the presence of co-overexpressed RIC157. Co-expressed GFP functions as cytoplasmic marker. Arrows indicate cytoplasmic strands. Microscopy pictures show maximum projections of at least 15 optical sections taken at 2 μm increments. Bar = 50 μm

To investigate the subcellular localization of RIC157, we fused GFP N-terminally to RIC157 and transiently expressed this construct in barley epidermal cells via biolistic transformation. As shown in Fig. 4A (upper panel), GFP-RIC157 expressed alone localizes to the cytosol, and a lack of GFP fluorescence in the nucleoplasm indicated that GFP-RIC157 is excluded from the nucleus. However, we repeatedly observed a strong fluorescent signal around the nucleus suggesting a potential affinity of RIC157 to the nuclear envelope/endoplasmic reticulum membrane or proteins associated to it (Suppl. Figure 3).

Since we found a direct protein-protein interaction between RIC157 and RACB, we checked the potential impact of RACB on the subcellular localization of RIC157 by transiently co-overexpressing GFP-RIC157 with various mCherry-tagged RACB forms (Fig. 4A). We detected GFP-RIC157 in the cytoplasm where it co-localized with mCherry fusions of RACB, DNRACB(T20N) and RACB(D121N). In contrast to that, a strong re-localization of GFP-RIC157 to the cell periphery was observed in the presence of mCherry-tagged CARACB(G15V) that likewise accumulated at this site. RACB, like other ROPs associates with the plasma membrane probably via its C-terminal posttranslational lipidations (Schultheiss et al. 2003; Weiß et al. 2022). Activated RACB then apparently recruits RIC157 to the cell periphery. Interestingly, the plasma membrane association of activated RACB and RIC157 appeared to be mutually promoted and locally stabilized (Fig. 4B). If expressed alone, mCherry-tagged activated RACB exhibited some cytoplasmic and nuclear localization which nearly completely disappeared in the presence of co-overexpressed RIC157.

To verify the localization of RIC157 at the plasma membrane, when recruited by activated RACB, we took advantage of a red fluorescent protein-tagged plasma membrane marker, pm-rk (Nelson et al. 2007), and analysed the potential co-localization of GFP-RIC157 with pm-rk in the presence of co-overexpressed non-tagged CARACB(G15V) (Suppl. Fig. S4). In the presence of activated RACB, fluorescence signals of GFP and mCherry unambiguously overlapped. This supported our interpretation that RIC157 is recruited by activated RACB to a plasma membrane-associated localization.

We further confirmed that the change in subcellular localization of RIC157 in the presence of activated RACB is dependent on the CRIB motif. GFP-tagged RIC157 lacking its CRIB motif localized similarly compared to intact RIC157 when expressed alone. However, it did not undergo a recruitment to plasma membrane-associated localizations neither in the presence of activated RACB nor any other RACB form (Suppl. Fig. S5).

Fluorescent proteins potentially have an impact on the functionality of the proteins to which they are fused due to conformational hindrances. In order to rule out that GFP impinges on the biological function of RIC157, we had also analysed the penetration success of *Bh* in barley epidermal cells overexpressing GFP-tagged RIC157 (Fig. 2C). The powdery mildew fungus benefited from the enhanced presence of GFP-RIC157, similar to untagged RIC157. This also supports that the observed subcellular localization of N-terminally tagged RIC157 represents the genuine localization of a functional RIC157 protein.

### RIC157 and RACB co-localize and accumulate at penetration site

**Fig. 5:**
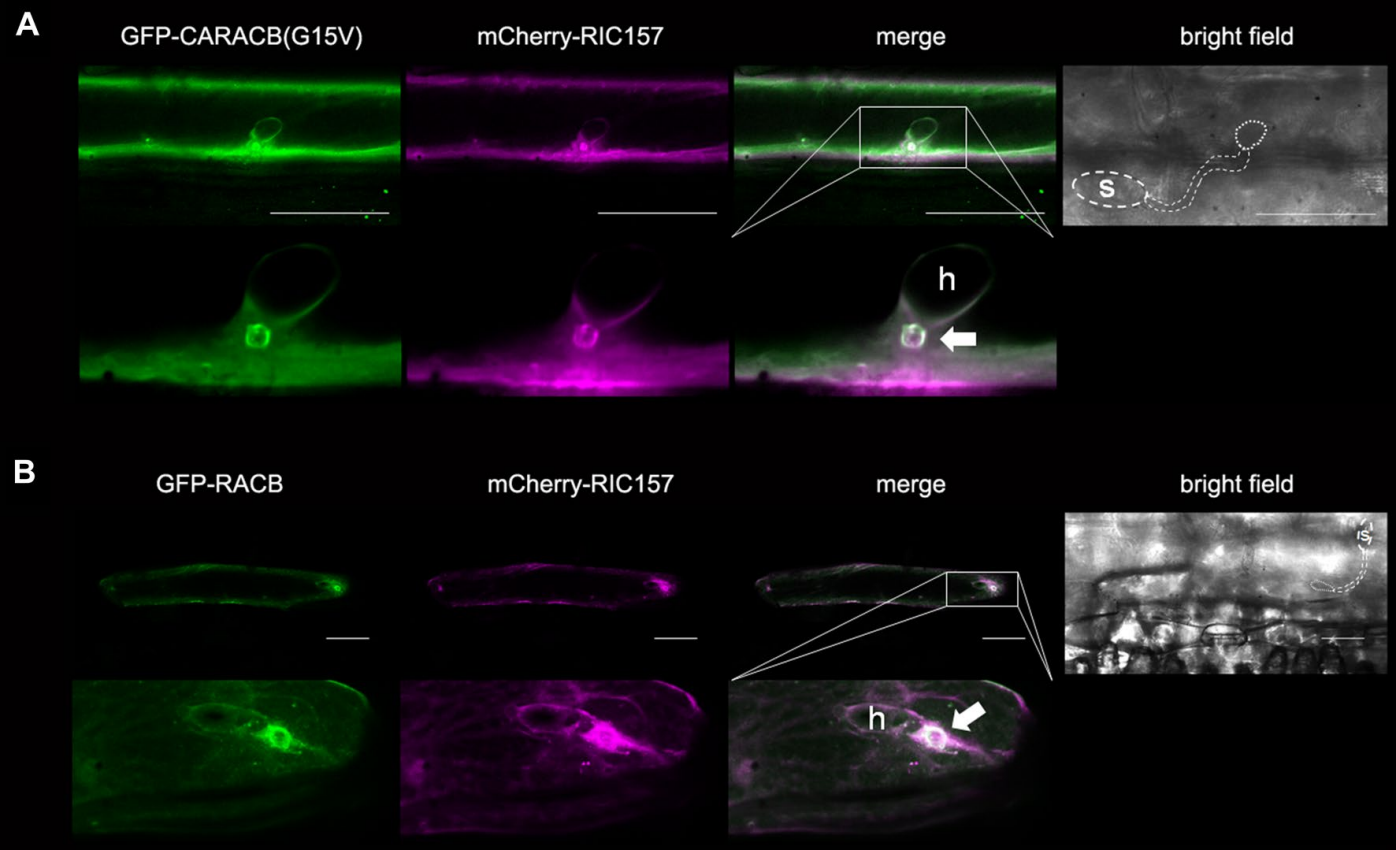
RIC157 is recruited to the penetration site where it colocalizes with activated RACB. Confocal laser scanning microscopy of epidermal cells 1d after transformation via particle bombardment and 18-24 h after inoculation with *Bh*. Cells in **A**, **B** show successful fungal penetration due to haustorium (h) formation. Area in white square is enlarged in lower panel. S = spore. Arrows indicate approximate position of the haustorial neck. Contrast of images was equally slightly enhanced. **A** Transient co-expression of GFP-CARACB(G15V) and mCherry-RIC157. Bar = 30 μm. **B** Transient co-expression of GFP-RACB and mCherry-RIC157. Bar = 50 μm

Co-localization experiments in unchallenged barley epidermal cells demonstrated a recruitment of RIC157 to the cell periphery in the presence of activated RACB. In order to investigate if both proteins similarly localize at a more specific subcellular site during the interaction with the powdery mildew fungus, we analysed the localization of fluorescently tagged transiently co-expressed RIC157 with constitutively activated CARACB(G15V) in barley epidermal cells 18–24 h after inoculation with *Bh*. As shown in Fig. 5A, fluorescent protein fusions of RIC157 and activated RACB accumulate close to the fungal penetration site, forming a ring outlining the neck of a developing haustorial initial, indicating a specific membrane-associated co-localization in epidermal cells that are successfully penetrated by the fungus. Interestingly, we observed a similar ring-like co-enrichment at the haustorial neck upon co-expression of fluorescent protein fusions of RIC157 and wildtype RACB (Fig. 5B). Since RIC157 interacts not with the presumably GDP-bound form of RACB, DNRACB(T20N) (Fig. 3), this suggests endogenous RACB activation and recruitment at the fungal entry site.

## Discussion

In the presence of *Bh*, high activity of the ROP protein RACB appears to be disadvantageous for barley. To date, however, our knowledge about RACB´s signaling surroundings of which the fungus takes advantage is still quite limited. Our studies now identify RIC157 as a RACB-interacting ROP-specific scaffold protein that might be exploited in barley epidermal cells by *Bh* to promote host cell accessibility.

### RIC proteins as scaffolds in RACB downstream signaling

In a signaling cascade, scaffold proteins are as vital for mediating the molecular response as upstream signaling hubs or downstream executors. Scaffolds establish not just a signaling hub-executor connection but they represent also the first branching point in the signaling cascade that eventually leads to specific events without possessing any kind of enzymatic activity themselves (Zeke et al. 2009; Good et al. 2011). ROPs function as signaling hubs and have been shown to be involved in loads of cellular processes, and they can regulate different, sometimes even antagonistic signaling pathways (Gu et al. 2005; Nibau et al. 2006; Feiguelman et al. 2018; Engelhardt et al. 2020). In case ROPs do not interact directly with downstream executors, RIC proteins and also ICRs/RIPs are considered bridging units, creating the scaffolds for specific branches of ROP signaling (Schultheiss et al. 2008; Craddock et al. 2012; Zhou et al. 2015; Hong et al. 2016; Feiguelman et al. 2018; Hoefle et al. 2020; McCollum et al. 2020). Besides RIC171 and RIC157, we identified another six proteins in barley that, by the above-mentioned definition, we consider RIC proteins. Potentially, there is a difference between monocots and dicots regarding the number of RIC proteins, which is slightly higher in Arabidopsis (Wu et al. 2001), suggesting a higher probability of either functional redundancies, diversification, or antagonistic partners in dicots (Gu et al. 2005). Redundancies between the barley leaf-expressed RIC proteins is, considering the low primary sequence conservation, hard to predict and not known yet. The partial similarities between leaf epidermis-expressed RIC157 and RIC171 regarding CRIB motif position and polybasic region close to the C-terminus indicate conserved functions in a similar signaling pathway.

### RIC157 increases susceptibility towards powdery mildew

In this study, we concentrated on leaf epidermis-expressed RIC157 and show, that the transient RIC157 overexpression leads to a significant increase in barley epidermal cell susceptibility towards powdery mildew infection (Fig. 2A). Thus, the fungus benefits from highly abundant RIC157. The RNA interference-mediated silencing of RIC157, however, did not lead to a higher resistance compared to control-treated cells (Fig. 2B). This could have different reasons. Although we have shown a significant reduction of GFP-RIC157 protein levels in the presence of the RIC157 RNAi silencing construct (Suppl. Fig. S2), it must be noted that RNAi-based silencing is never 100% efficient. Remaining endogenous RIC157 transcript levels might be sufficient to allow for control level penetration efficiencies. Additionally, the protein turnover rate of RIC157 is unknown, meaning it is still possible that RIC157 protein, expressed before transient transformation with the RNAi silencing construct, is present and sufficiently abundant to support penetration. Another very important point that needs to be stressed is the function of ROPs as signaling hubs (Nibau et al., 2006). The signaling via RIC157 is likely not the only RACB-regulated path that leads to susceptibility. Shutting down the particular RACB-RIC157 route probably still leaves other RACB signaling branches functional, which are potentially involved to a certain extent in RACB-mediated susceptibility as well. Enhanced resistance towards fungal infection was, however, achieved once the signaling hub was removed or switched off by silencing RACB or by transient overexpression of its negative regulator MAGAP1 (Schultheiss et al., 2002; Hoefle et al., 2011).

### RIC157 and activated RACB interact and mutually support a specific localization

We have demonstrated that RIC157 can interact directly with RACB in yeast and *in planta* (Fig. 3). In barley epidermal cells, RIC157 showed interaction with activated RACB but not with the dominant negative form. The observed interaction in yeast or *in planta* between RIC157 and RACB(D121N) could be explained by different experimental setups and/or physiological cell conditions. G-proteins with this particular mutation have been observed previously to display either dominant-negative or constitutively activated properties (Cool et al., 1999). RACB(D121N) has a presumed intrinsic lower nucleotide affinity, however in yeast and in barley epidermal cells this mutant might adopt a conformation resembling the activated RACB by being predominantly GTP bound.

The subcellular *in planta* RACB-RIC157 interaction site, as seen in FLIM-FRET and co-expression experiments, appeared to be at the cell periphery and plasma membrane-associated. This seems conclusive, since RIC157 exclusively interacts with activated RACB. Activated ROPs in turn are allegedly associated with negatively charged phospholipids in plasma membrane domains, and this is further promoted via posttranslational prenylation and S-acylation (Yalovsky 2015; Platre et al. 2019). In this work and previously (Schultheiss et al. 2003), it has been shown that fluorescent protein-tagged CARACB(G15V) transiently over-expressed alone localizes preferentially to both cell periphery and cytoplasm. Plasma membrane association of activated ROPs might require additional stabilization by downstream signaling interactors. This is supported by our findings regarding CARACB(G15V) co-expressed with RIC157 (Fig. 4B) that substantially promotes the plasma membrane localization of activated RACB. While the N-terminal part of RIC157 containing the CRIB domain binds to RACB, a polybasic amino acid stretch in the C-terminal part of RIC157 is possibly responsible for connecting non-covalently to anionic plasma membrane phospholipids, thereby stabilizing the plasma membrane association of RACB. On the other hand, linking to anionic phospholipids is not sufficient, though, for RIC157 localizing to the plasma membrane. Expressed alone, fluorescence-tagged RIC157 localizes essentially to the cytoplasm and is excluded from the nucleus (Fig. 4A; Suppl. Figure 3). In the presence of activated RACB, RIC157 undergoes a CRIB motif-dependent relocalization from the cytoplasm to the cell periphery/plasma membrane. A similar recruitment to the cell periphery has been observed with other proteins that directly interact with activated RACB (Schultheiss et al. 2008; Huesmann et al. 2012; McCollum et al. 2020). This supports the hypotheses that RACB activation precedes the recruitment of interaction partners and downstream RACB signaling is initiated at the plasma membrane. The latter point is further backed up by a cytoplasm localised RACB mutant version lacking the C-terminal CSIL motif required for prenylation that is inactive in promoting susceptibility to fungal penetration (Schultheiss et al. 2003; Weiß et al. 2022).

With regard to RACB’s and RIC157’s capability to support fungal infection, the recruitment of RIC157 to the cell periphery becomes even more interesting. Our data suggest a recruitment of activated RACB and RIC157 to the fungal penetration site (Fig. 5). The subcellular co-localization site in particular appears to be a ring-like structure surrounding the haustorial neck and penetrated cell wall apposition. This co-localization of RACB and fungal infection-supporting RACB-interactors is not exclusive to RIC157. RIC171 and RIPb, two proteins also considered scaffolds in RACB signaling, likewise co-localize with RACB at the haustorial neck (Schultheiss et al. 2008; Hückelhoven and Panstruga 2011; McCollum et al. 2020). Future experiments may show whether subcellular co-enrichment of RACB and RACB-interacting proteins indicates a specific lipid composition in the haustorial neck, which then helps in recruiting activated ROPs or membrane domains of high ROP activity due to local GEF activity or both. Additionally, it could indicate an exclusion of ROPs from further lateral diffusion into the EHM, which was suggested to be controlled at the haustorial neck for Arabidopsis (Koh et al., 2005).

### RIC157 function and susceptibility

RIC157 transiently overexpressed in barley epidermal cells localizes to the cytoplasm and enhances fungal penetration efficiency. We assume, though, that transiently overexpressed RIC157 promotes RACB-mediated susceptibility upon recruitment to the cell periphery by endogenously present activated RACB. Only a small fraction of overexpressed RIC157 is possibly recruited to the cell periphery without concomitant co-overexpression of activated RACB. This means that such a minute fluorescence localization change is hard to detect in our experimental setup, but we want to stress that we do not assume RIC157 promoting susceptibility to powdery mildew from a solely cytoplasmic site. Additionally, overexpressed RIC157 potentially outtitrates other interaction partner of activated RACB, including negative regulators, which leads to all endogenous RACB proteins interacting exclusively with RIC157, thereby being stabilized in its signaling active conformation at the plasma membrane. Indeed, in front of a cytoplasmic background, RIC157 is to a certain extent also marginally visible at the cell periphery without co-expressing CARACB(G15V), and this possibly reflects a partial recruitment also by endogenous ROPs (Fig. 4A). Indeed, RIC157 and the negative RACB-regulator MAGAP1 both possess a CRIB motif for RACB interaction, and also MAGAP1 can be observed at the haustorial neck of penetrated cells (Hoefle et al. 2011). It is tempting to speculate that positive and negative regulators of RACB signaling compete for RACB binding via their CRIB motif. Hence, overexpression of a positive regulator such as RIC157 may shift the balance into a more signaling-active situation.

The phylogenetic relation between barley RIC157 and *Arabidopsis thaliana* RIC10 and RIC11 (Suppl. Figure 1) does not necessarily indicate similar functions of both proteins. Besides *At*RIC10 and *At*RIC11, for which a biological function is unknown to date, RIC157 also shares some more distant similarity to *At*RIC1, *At*RIC3 and *At*RIC7 in clade II, for which an involvement in cytoskeleton organization has previously been demonstrated. Of these three *Arabidopsis* RIC proteins, *At*RIC1 appears to be an interesting candidate from which a RIC157 function could be deduced. *At*RIC1 has been shown to interact with *At*ROP6 to activate the p60 subunit of Katanin (*At*KTN1), a microtubule-severing enzyme (Lin et al. 2013), as opposed to the interaction with *At*ROP2 that negatively regulates the action of *At*RIC1 on microtubules (Fu et al. 2005). The microtubule-severing activity of *At*KTN1 might even be regulated by *At*ROP2, *At*ROP4 and *At*ROP6 (Ren et al. 2017) via *At*RIC1. We observed a role of RACB or RACB-interacting proteins in polar cell development and microtubule organization (Hoefle et al. 2011; Huesmann et al. 2012; Scheler et al. 2016; Nottensteiner et al. 2018). It is, however, unknown how RACB might influence cytoskeleton organisation or polar membrane trafficking. It still remains to be seen whether barley RIC157 also fulfils a *At*RIC1-like regulatory role in organising microtubules. Regarding the subcellular localization there are, however, clear differences: Barley RIC157 localizes predominantly to the cytoplasm, *At*RIC1 associates with microtubules (Fu et al. 2005). However, when microtubule arrays in penetrated barley epidermal cells were compared with attacked but non-penetrated ones, penetration success was strongly associated with parallel non-polarized microtubule arrays in the cell cortex and a diffuse or depleted microtubule structure at the haustorial neck (Hoefle et al. 2011). *At*RIC3 has been shown to be involved in the pollen tube growth process, where its function leads to actin disassembly in a ROP1-dependent manner upon calcium influx into the cytoplasm (Gu et al. 2005; Lee et al. 2008). Likewise, *At*RIC7 was recently reported to influence vesicle trafficking in stomata, resulting in the suppression of an elevated stomatal opening after *At*ROP2-dependent inhibition of the exocyst complex *via At*Exo70B1 (Hong et al. 2016). Both F-actin organization and exocyst function are important in penetration resistance to *Bh* (Opalski et al. 2005; Miklis et al. 2007; Ostertag et al. 2013). Therefore, the discovery of RIC157 as a susceptibility-promoting factor may pave the way to a better understanding of ROP-steered processes that are pivotal for fungal invasion into barley epidermal cells. The challenge will be to find the downstream factors that RIC157 activates and to understand how RIC157 interacts with RACB in the presence of several other RACB interactors. We assume that simultaneously expressed diverse RICs and ICR/ROP proteins could form a cooperative network for orchestrating F-actin, microtubule and membrane organization at the site of fungal entry.

## Electronic supplementary material

Below is the link to the electronic supplementary material.

Supplementary Material 1

## Data Availability

The datasets generated during this work are available from the corresponding author on reasonable request.
