# Supplementary material for: Barley RIC157, a potential RACB scaffold protein, is involved in susceptibility to powdery mildew
Source: Plant Mol Biol. 2022 Dec 23;111(4-5):329–44. doi: 10.1007/s11103-022-01329-x (PMC10090020; doi:10.1007/s11103-022-01329-x)
Supplement: Supplementary file 1 — Supplementary Material 1 [file 11103_2022_1329_MOESM1_ESM.pdf]

Barley RIC157, a potential RACB scaffold protein, is involved in susceptibility to powdery mildew

Plant Molecular Biology

Stefan Engelhardt, Adriana Trutzenberg, Michaela Kopischke, Katja Probst, Christopher McCollum, Johanna Hofer and Ralph Hückelhoven#

Phytopathology, School of Life Science Weihenstephan, Technical University of Munich, Emil-Ramann-Str.2, 85354 Freising, Germany

#Corresponding author: hueckelhoven@tum.de

Supplements

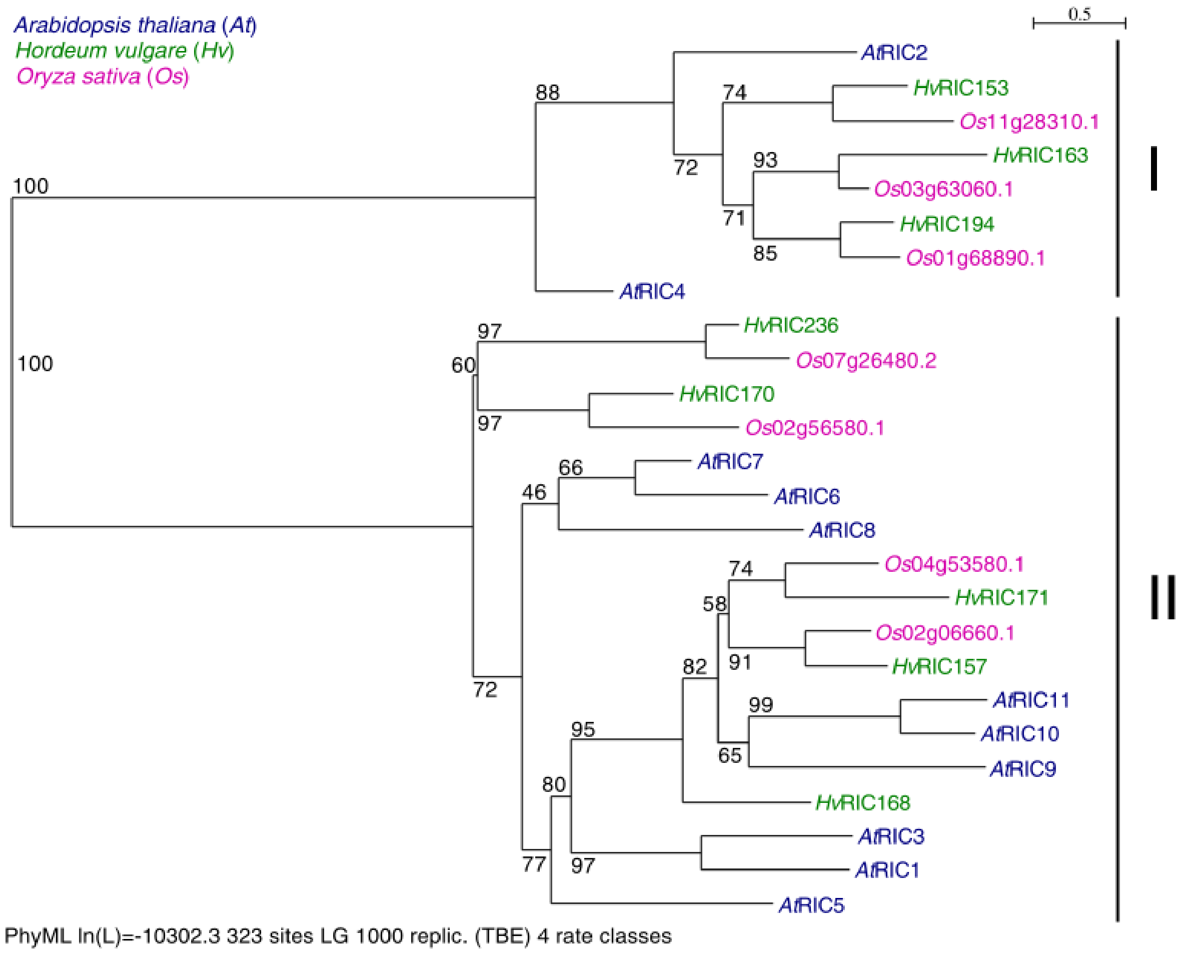

Suppl. Fig. S1: Phylogenetic analysis of RIC proteins from barley (*Hordeum vulgare*, *Hv*), *Arabidopsis thaliana* (*At*) and rice (*Oryza sativa*, *Os*). Maximum likelihood calculation using PhyML LG model following a muscle alignment via SeaView (Version 5.0.4.). Indicated values (in %) represent bootstrap values of 1000 repetitions, with the scale bar specifying the mean number of amino acid substitutions per site

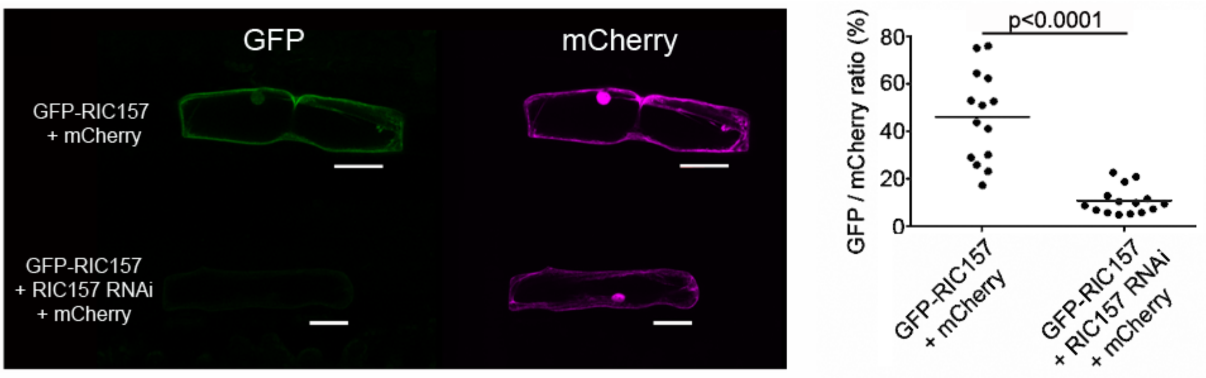

Suppl. Fig. S2: RNA interference-mediated silencing efficiency. Epidermal cells of 7-days-old barley primary leaves were transiently transformed via particle bombardment with overexpression constructs of a GFP fusion of RIC157 alone or together with the RNAi silencing construct. A construct to express cytosolic mCherry was simultaneously co-delivered for transformation efficiency and fluorescence quantification purposes. Microscopy images are maximum projections of at least 15 optical sections taken at 2µm increments. Bar = 50µm. Each graph shows the mean of GFP fluorescence as percentage of mCherry fluorescence per transformed cell (whole cell area was taken as region of interest for measuring fluorescence intensity). Each dots represent a single cell. Statistical significance calculated with t-test

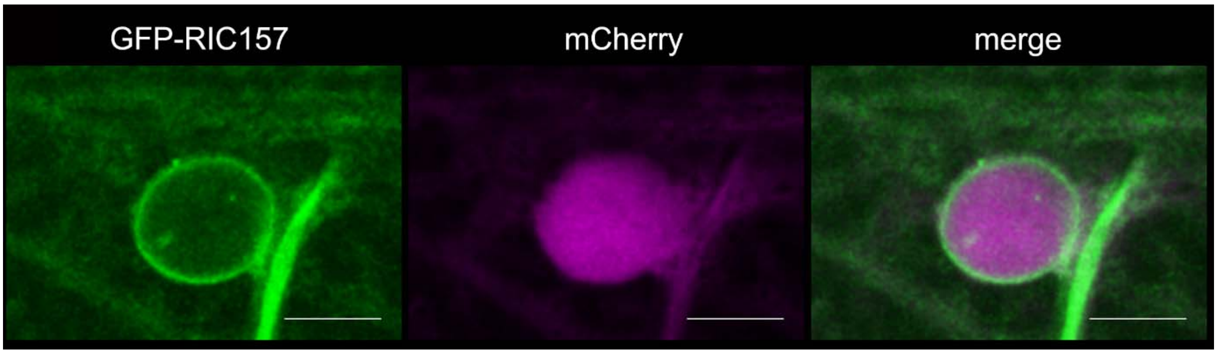

Suppl. Fig. S3: RIC157 does not localize into the nucleus. Confocal laser scanning microscopy of barley epidermal cells 1d after transformation via particle bombardment. GFP-RIC157 localises to the cytoplasm and is excluded from the nucleus. Co-expressed mCherry functions as cytoplasmic and nucleoplasmic marker. Microscopy pictures show a single optical section. Bar = 10µm

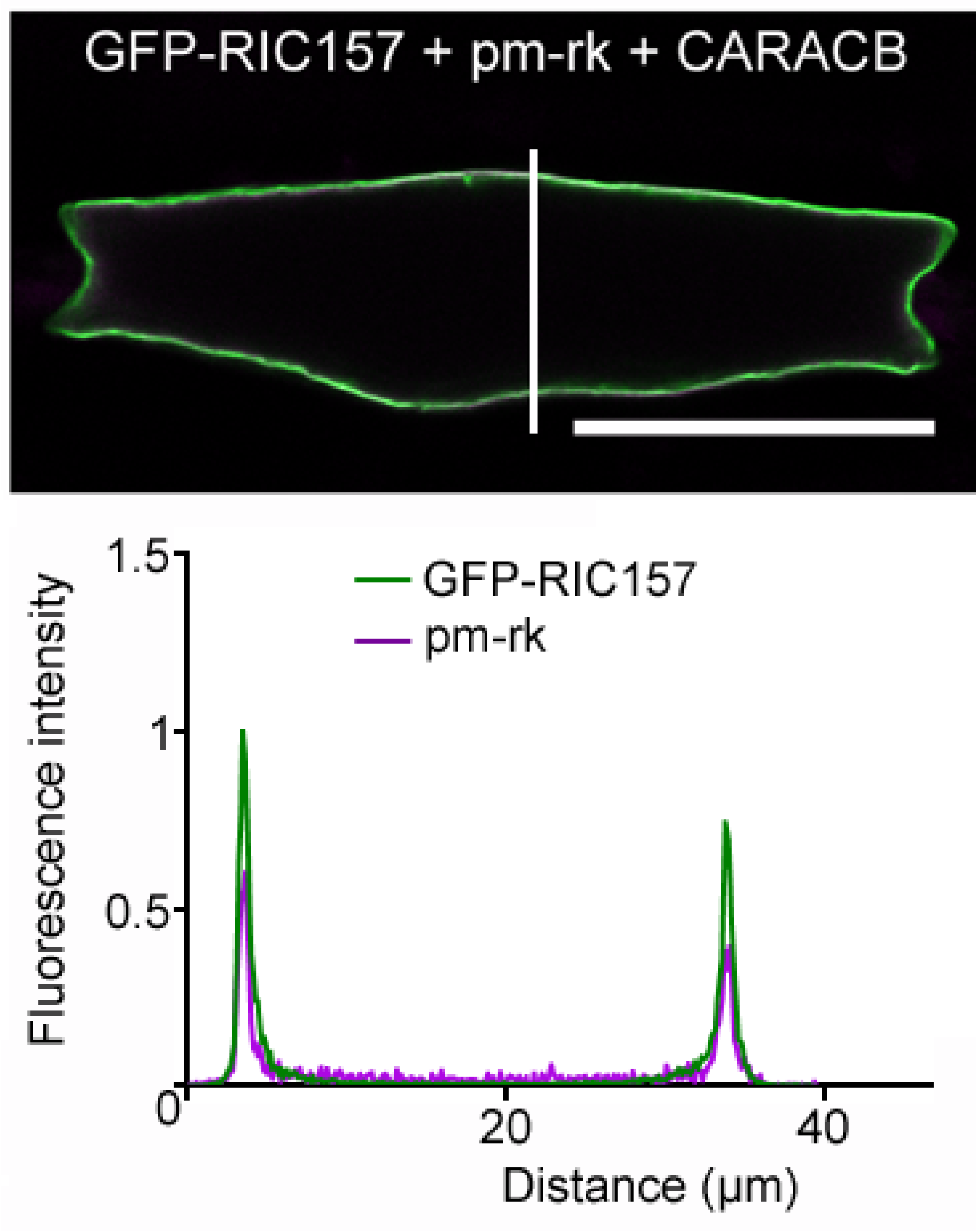

Suppl. Fig. S4: RIC157, recruited to the cell periphery by activated RACB, colocalises with plasma membrane marker. Confocal laser scanning microscopy of a representative barley epidermal cell 1d after transient transformation via particle bombardment to express GFP-RIC157, red-fluorescent plasma membrane marker pm-rk (Nelson et al. 2007) and non-tagged CARACB(G15V). The microscopy picture (upper panel) shows a single optical section to confirm specific co-localisation of GFP-RIC157 with plasma membrane marker pm-rk. In the lower panel, an overlay analysis of both fluorescences is shown via graphical visualization of fluorescence signal intensities measured over a region of interest (white vertical line in microscopy picture). Bar = 50µm

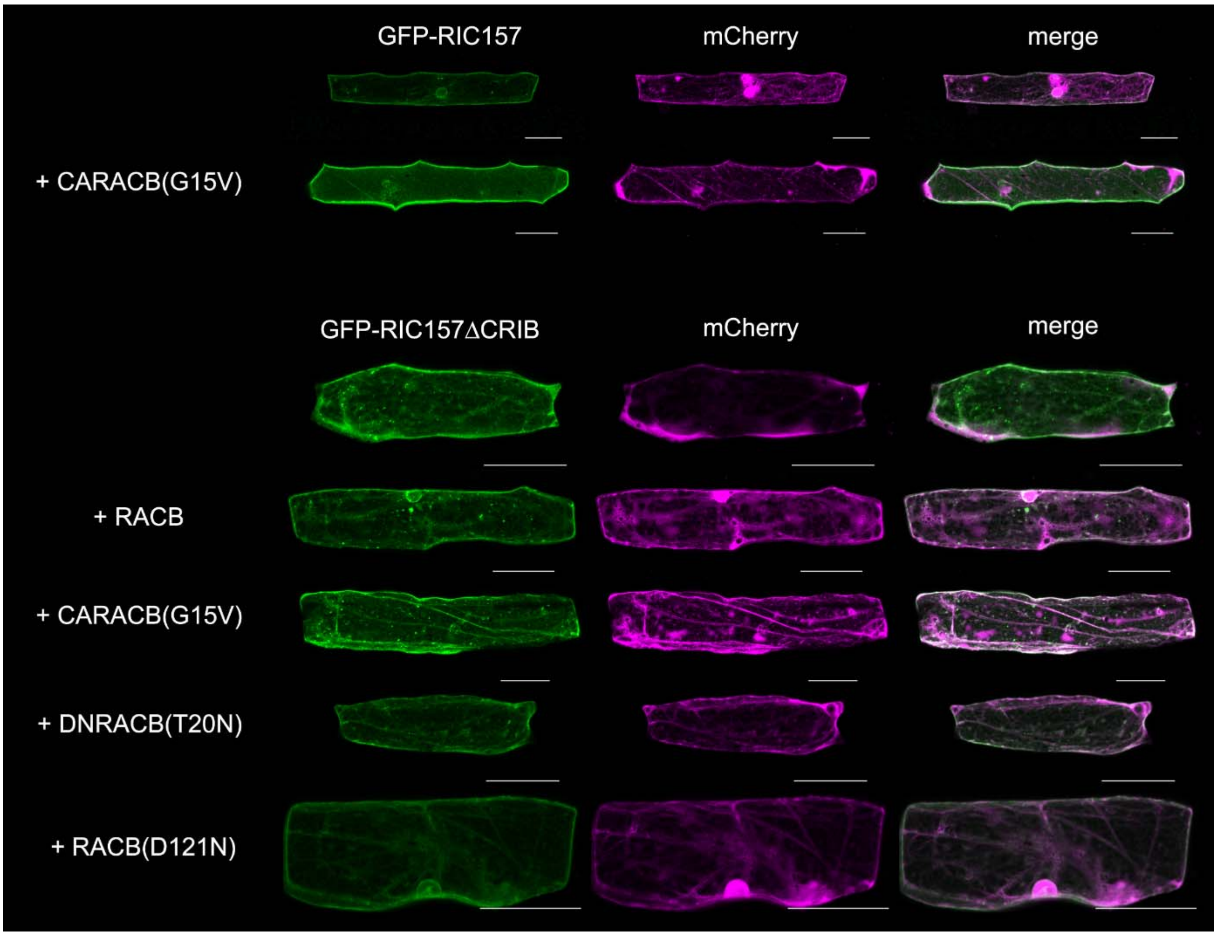

Suppl. Fig. S5: RIC157 recruitment to the plasma membrane by activated RACB is CRIB motif-dependent. Confocal laser scanning microscopy of barley epidermal cells 1d after transformation via particle bombardment. Both GFP-RIC157 and GFP-RIC157ΔCRIB localise to the cytoplasm, but only GFP-RIC157 (upper panel) is recruited to the cell periphery by co-overexpressed CARACB(G15V), GFP-RIC157ΔCRIB localization is not changed when co-expressed with either RACB form. Co-expressed mCherry functions as cytoplasmic marker. Microscopy pictures show maximum projections of at least 15 optical sections taken at 2μm increments. Bar = 50μm

Suppl. Table 1: Primers used in this study

Primer name	Gene (construct)	Sequence
RIC157_GW_for	*RIC157*	5‘-AAAAAGCAGGCTCACAAATGGCGGTAAAGATGAAGGG-3‘
RIC157_GW_rev+STOP	*RIC157*	5‘-AGAAAGCTGGGTCACCGCCTCCGGATCAGACGACTCGAACCCCTCTTTGC-3‘
RIC157delCRIB_for	*RIC157ΔCRIB*	5‘-GCTCAAAAGGAGCATGAGATGGAATTGGGCACCAGTGACACATC-3‘
RIC157delCRIB_rev	*RIC157ΔCRIB*	5‘-CACTGGTGCCCAATTCCATCTCATGCTCCTTTTGAGC-3‘
attB1	*attB1*	5‘-GGGGACAAGTTTGTACAAAAAAGCAGGCTCACAA-3‘
attB2	*attB2*	5‘-GGGGACCACTTTGTACAAGAAAGCTGGGTCACCG-3‘
RIC157_RNAi_NotI_for	*RIC157 RNAi*	5‘-AATTGCGGCCGCAAGATGAAGGGAATCTTCAAAGGGC -3‘
RIC157_RNAi_XbaI_rev	*RIC157 RNAi*	5‘-AATTTCTAGAACGCCGTCGCGAAGGAGGCCCTCGACC -3‘
RIC157_RNAi_EcoRI_for	*RIC157 RNAi*	5‘-AATTGAATTCTTGGGCACCAGTGACACATCTCC-3‘
RIC157_RNAi_EcoRI_rev	*RIC157 RNAi*	5‘-AATTGAATTCTTCCATCTCATGCTCCTTTTGAGC-3‘
RIC157_BamHI_for	*RIC157*	5‘-AATTGGATCCATGGCGGTAAAGATGAAGGGAATC-3‘
RIC157_KpnI_rev	*RIC157*	5‘-AATTGGTACCCTAGACGACTCGAACCCCTCTTTGC-3‘
RIC157_for	*RIC157*	5‘-ATGGCGGTAAAGATGAAGG-3‘
RIC157_rev	*RIC157*	5‘-GACGACTCGAACCCCTCTTTGC-3‘
RACB_D121N_fw	*DNRACB(D121N)*	5‘-CTCGTGGGAACAAAGCTTAATCTTCGAGATGACAAG-3‘
RACB_D121N_rv	*DNRACB(D121N)*	5‘-CTTGTCATCTCGAAGATTAAGCTTTGTTCCCACGAG-3‘
RACB_GW_for	*RACB*	5‘-AAAAAGCAGGCTCACAAATGAGCGCGTCCAGGTTCATAAAGTGC-3‘
RACB_GW_rev	*RACB*	5‘-AGAAAGCTGGGTCACCGCCTCCGGACAAGATGGAGCAAGCCCCCC-3‘
HvUBC2_fwd	*Ubiquitin conjugating enzyme 2*	5‘-TCTCGTCCCTGAGATTGCCCACAT-3‘
HvUBC2_rev	*Ubiquitin conjugating enzyme 2*	5‘-TTTCTCGGGACAGCAACACAATCTTCT-3‘
delCSIL_for	*RACBΔCSIL*	5‘-GAAGAAAAAGGCGCAGAGGGGGGCTTGATCCATCTTGTAGTCCGGAGGCGGTG-3‘
delCSIL_rev	*RACBΔCSIL*	5‘-CACCGCCTCCGGACTACAAGATGGATCAAGCCCCCCTCTGCGCCTTTTTCTTC-3‘
GW_RfA_mCherry-F	*mCherry*	5‘-GCTGTACAAGATCACAAGTTTGTACAAAAAAGCTG-3‘
GW_RfA_meGFP-F	*meGFP*	5‘-GCTGTACAAAATCACAAGTTTGTACAAAAAAGCTG-3‘
GW_RfA_Xba-R	*RfA*	5‘-TGCCTGCAGGTCGACTCTAGAATCACCACTTTGTACAAGAAAGCTG-3‘
GW_Xba_RfB-F	*RfB*	5‘-GGTACCCGGGGATCCTCTAGAATCAACAAGTTTGTACAAAAAAGCT-3‘
GW_RfB-R	*RfB*	5‘-TGCTCACCATATCAACCACTTTGTACAAGAAAGCT-3‘
meGFP-STP-F	*meGFP*	5‘-AGTGGTTGATATGGTGAGCAAGGGCGAGG-3‘
mCherry-STP-F	*mCherry*	5‘-AGTGGTTGATATGGTGAGCAAGGGCGAGG-3‘
XFP-noSTP_Xba-F	*XFP*	5‘-GGTACCCGGGGATCCTCTAGAATGGTGAGCAAGGGCGAGG-3‘
XFP-noSTP-R	*XFP*	5‘-AACTTGTGATCTTGTACAGCTCGTCCATGCC-3‘
meGFP-noSTP-R	*meGFP*	5‘-AACTTGTGATTTTGTACAGCTCGTCCATGCC-3‘
mCherry-STP_Xba-R	*mCherry*	5‘-TGCCTGCAGGTCGACTCTAGATTACTTGTACAGCTCGTCCATGCC-3‘

meGFP-STP_Xba-R	*meGFP*	5‘-TGCCTGCAGGTCGACTCTAGATTATTTGTACAGCTCGTCCATGCC-3‘
